# Cost of care pathways before and after appropriate and inappropriate transfers to the emergency department among nursing home residents: results from the FINE study

**DOI:** 10.1186/s12877-024-04946-x

**Published:** 2024-04-19

**Authors:** E. Gombault-Datzenko, N. Costa, M. Mounié, N. Tavassoli, C. Mathieu, H. Roussel, J. M. Lagarrigue, E. Berard, Y. Rolland, L. Molinier

**Affiliations:** 1 Present address: Department of Medical Information (DIM), Toulouse University Hospital, 2 rue Viguerie, Toulouse Cedex 9, 31059 France; 2 INSERM, UMR 1295, Toulouse, France; 3 Gérontopôle, Toulouse University Hospital, Toulouse, France; 4 CREAI-ORS Occitanie, Toulouse, France; 5 CNAM, DRSM Occitanie, 2 rue Georges Vivent, Toulouse, 31082 France; 6 MSA Midi-Pyrénées Nord, 180 Avenue Marcel Unal, Montauban, 82000 France; 7 Department of Epidemiology, University Hospital of Toulouse, 37 Allées Jules Guesde, Toulouse, 31000 France

**Keywords:** Cost, Economics, Long-term care unit, Nursing home, Transfer to emergency department

## Abstract

**Background:**

Transfers of nursing home (NH) residents to the emergency department (ED) is frequent. Our main objective was to assess the cost of care pathways 6 months before and after the transfer to the emergency department among NH residents, according to the type of transfer (i.e. appropriate or inappropriate).

**Methods:**

This was a part of an observational, multicenter, case-control study: the Factors associated with INappropriate transfer to the Emergency department among nursing home residents (FINE) study. Sixteen public hospitals of the former Midi-Pyrénées region participated in recruitment, in 2016. During the inclusion period, all NH residents arriving at the ED were included. A pluri-disciplinary team categorized each transfer to the ED into 2 groups: appropriate or inappropriate. Direct medical and nonmedical costs were assessed from the French Health Insurance (FHI) perspective. Healthcare resources were retrospectively gathered from the FHI database and valued using the tariffs reimbursed by the FHI. Costs were recorded over a 6-month period before and after transfer to the ED. Other variables were used for analysis: sex, age, Charlson score, season, death and presence inside the NH of a coordinating physician or a geriatric nursing assistant.

**Results:**

Among the 1037 patients initially included in the FINE study, 616 who were listed in the FHI database were included in this economic study. Among them, 132 (21.4%) had an inappropriate transfer to the ED. In the 6 months before ED transfer, total direct costs on average amounted to 8,145€ vs. 6,493€ in the inappropriate and appropriate transfer groups, respectively. In the 6 months after ED transfer, they amounted on average to 9,050€ vs. 12,094€.

**Conclusions:**

Total costs on average are higher after transfer to the ED, but there is no significant increase in healthcare expenditure with inappropriate ED transfer. Support for NH staff and better pathways of care could be necessary to reduce healthcare expenditures in NH residents.

**Trial registration:**

clinicaltrials.gov, NCT02677272.

**Supplementary Information:**

The online version contains supplementary material available at 10.1186/s12877-024-04946-x.

## Background

The number of people aged 60 years and older worldwide is increasing. There were 1 billion in 2019 and this number will increase to 2.1 billion by 2050 [1]. This increase is occurring at an unprecedented pace and requires adaptations of societal structures across all sectors.

In France, there were 17 million people aged 60 years or older in 2018. This number will reach 24 million by 2060 [2]. In 2016, of the French population, 728,000 elderly people lived in nursing homes (NHs), i.e. 10% of people aged 75 years or older and one third of those aged 90 years or older [3]. NHs cater for people aged 60 and over, with varying degrees of dependency. They may be public, private or associative (private not-for-profit). The way they are organized, the staff present (coordinating physician, nurses…) and the charges vary according to the care offered to residents.

Around 50% of NH residents are hospitalized at least once per year in France, and there is an intense flow between NHs and emergency departments (EDs) [4, 5], as is also the case in other countries (Australia, Ireland, Canada, etc.) [6–8]. French and international observational studies have shown that 50% of NH residents per year are transferred to an ED [9]. These transfers are often inappropriate (about 40% of cases) and costly [10–13]. Moreover, when the transfer to the ED is inappropriate (i.e. not a health emergency, normal vital signs), the benefit / risk balance for the patient is often unfavorable, with a high risk of confusion and functional decline [14, 15].

For the French health insurance (FHI) in 2016, the median annual care cost per NH resident was 14,375€, with 12% for outpatient costs [16]. This cost was major and increased with the resident’s level of dependency and comorbidities. A few studies suggested that reducing inappropriate hospitalizations of NH residents could lead to lower costs [17–19]. We can hypothesize that inappropriate transfer is a sign of non-integrated care of elderly people before arrival in the emergency room. Then, inappropriate transfer to the ED could be associated with increased healthcare utilization and associated expenditures 6-months before and after the transfer (in- and outpatient costs).

The main objective of the current study was to assess the cost of care pathways 6 months before and after the transfer to the emergency department among NH residents, according to the type of transfer (i.e. appropriate or inappropriate). The second objective was to explore factors associated with healthcare utilization’s costs among NH residents.

## Methods

### Setting, design and population

This study is a part of the Factors associated with INappropriate transfer to the Emergency department among nursing home residents (FINE) study [20]. The FINE study, which is an observational, multicenter, case-control study (clinicaltrials.gov, NCT02677272), initially aimed to identify factors associated with inappropriate transfers to the ED, by comparing resident and NH characteristics, as well as the circumstances upon transfer to the ED.

Sixteen public hospitals (from among 25 in the former Midi-Pyrénées region in the southwest of France) participated in recruitment for the FINE study, from January 2016 to December 2016 (12 months). During the inclusion period, all residents arriving at the ED from an NH were included, the only inclusion criterion being arrival from an NH.

A team of experts (one geriatrician, a family doctor, an emergency doctor and a pharmacist) defined appropriate/inappropriate transfers to the ED during a face-to-face meeting using a standardized approach. Charts for each resident were reviewed by the team and three criteria characterizing inappropriate transfer were discussed: the lack of somatic or psychiatric emergency conditions, the presence of palliative care known before and advanced directives of non-hospitalization. Indeed, in these 3 clinical situations, patients should be managed differently, without being transferred to the emergency department, with no increased health risk. A patient in palliative care no longer requires emergency care, and should therefore not be transferred to an emergency department. Similarly, a patient who has formulated advance directives of non-hospitalization should not be transferred to a hospital. The method has been previously reported in detail [11].

### Cost estimates

Healthcare costs were assessed from the FHI perspective. Direct medical and non-medical costs were included in this study. Direct costs corresponded to hospitalization costs, outpatient costs (i.e. visits and medical acts [imaging and other preventive exams, diagnostic exams and curative acts], paramedical acts [nurse, physical therapist and speech therapist]), medications and medical equipment costs. Non-medical costs included transportation costs. Costs were estimated by multiplying the number of units used for each resource with the corresponding unit cost.

The consumption of healthcare resources was retrospectively gathered from the FHI database. It contains fee-for-service claims for in- and outpatient medical services supplied to 80% of the residents of France (belonging to the general worker’s insurance scheme and agricultural workers insurance scheme) [21]. The remaining 20% of French residents are covered by special subdivisions of the French social healthcare system, depending on their job and their fee-for-services claims are not available in the FHI database. Administrative data corresponding to the first name, last name, birthdate, place of residence and sex were recorded for patients who live in an NH located in the Midi-Pyrénées region.

Inpatient stays were valued using per diem costs. Outpatient care, which includes visits, medical and paramedical acts, medications, medical equipment and transportation, was valued using the tariffs reimbursed by the FHI. In particular, transportation, visits and paramedical acts were valued using the French General Nomenclature of Professional Acts [22]. Medical acts were valued using the French Common Classification of Medical Acts [23], except for laboratory tests, for which valuation was based on the Nomenclature of Biological Acts [24]. Medical equipment was valued using the List of Reimbursable Products and Services [25]. Medications were valued using retail prices, and their reimbursement was present in the database only for NHs without a pharmacy for internal use (PIU) [26].

For all these fees, we applied the corresponding reimbursement rate and we subtracted, if necessary, the medical deductible, which is due by the patient and not reimbursed by the FHI. Costs were expressed in 2015–2016 euros and were recorded over a 6-month period before and after the transfer to the ED.

### Other variables

Variables useful for the cost analysis collected during the FINE study were:

- For the characteristics of the residents: sex, age, comorbidities using the Charlson score (score from 0 to 37), level of dependence using the Katz Activities of Daily Living (ADL) scale (score from 0 “total dependency” to 6 “autonomy”), vital status, if the resident is receiving palliative care and the presence of advanced directives for non-hospitalization,
- For the characteristics of the NH: location (French department), NH with pharmacy for internal use, number of beds, distance (in kilometers) to the ED and available NH staff (presence of a coordinating physician or a geriatric nursing assistant),
- For the characteristics of the transfer: season of transfer (January to March = winter, April to June = spring, etc.) and post-transfer destination (hospitalization, back at NH or death).

### Statistical analysis

Patients were divided into two groups according to ED transfer: appropriate or inappropriate. Resident, NH and ED transfer characteristics were described using means and standard deviation for continuous variables and using frequencies and percentages for categorical variables. These two groups were also compared with a bivariate analysis (Mann-Whitney test for continuous variables, Chi2 test or Fisher’s exact test for categorical variables). Regarding the analysis of the main objective: for total direct costs, hospitalizations costs and outpatient costs, monthly mean costs and their 95% confidence intervals were calculated for 6 months before and after the transfer to the ED, in the two groups, and compared using the Wilcoxon test. Regarding the analysis of the secondary objective: A generalized linear mixed model (GLMM) with gamma distribution and log link was implemented to adjust monthly cost variation on confounding factors. GLMM allows the correlation within the longitudinal data to be taken into account [27]. Covariates used in the model were type of transfer (appropriate vs. inappropriate), sex, age (3 categories : < 85years old/ 85–90 years old/ > 90 years old), Charlson score (2 categories: 0–2 / ≥3), period before or after transfer, season (2 categories: winter-spring/ summer-autumn, i.e. a year divided into two semesters), death (month of death and month before, or not) and presence of a coordinating physician or a geriatric nursing assistant in the NH. We had to reduce the number of categories for some variables, in order to make the analysis possible with a sufficient sample size. A GLMM was also developed over two separate periods: before and after transfer to the ED. All statistical analyses were performed using the R software (version 3.5.3).

## Results

### Characteristics of the studied population

Data were available for 616 patients in the FHI database (59.4% of patients included in the FINE study). Among these, 132 (21.4%) had an inappropriate ED transfer (see the flow chart in Supplementary Figure S1).

Table 1 shows the descriptive characteristics of residents, of NHs and of ED transfer according to the appropriateness of the transfer. Residents with an inappropriate ED transfer were younger (mean age difference: -1.53 years, *p* = 0.024) and were more often under palliative care (+ 10%, *p* < 0.001) than residents with an appropriate ED transfer. The post-transfer destination are also significantly different between two groups: more hospitalizations with appropriate ED transfer (+ 27%, *p* < 0.001) and more returns to the NH with inappropriate ED transfer (+ 27%, *p* < 0.001) were observed. The characteristics of our population (616 residents) were similar to those of the FINE population not found in FHI database (421 residents) (see Supplementary Table S2, no significant differences, except for age in the inappropriate ED transfer group). During the 6 months after ED transfer, 208 residents died, 45 (34%) in the inappropriate ED transfer group and 163 (34%) in the appropriate ED transfer group.

**Table 1:** Population characteristics at emergency transfer date and comparison between inappropriate and appropriate ED transfer groups

	All *N* = 616	Inappropriate ED transfer *N* = 132	Appropriate ED transfer *N* = 484	Statistical test	*P*-value
**Characteristics of the residents**					
Sex					
* Female*	425 (69%)	86 (65%)	339 (70%)	Chi2	0.282
Age (mean, SD)	87 (7.29)	85.81 (7.97)	87.34 (7.06)	Mann-Whitney	**0.024**
Charlson score					
* 0*	55 (9%)	14 (10%)	41 (8%)	Chi2	0.561
* 1 to 2*	269 (44%)	58 (44%)	211 (44%)		
* 3 to 4*	184 (30%)	34 (26%)	150 (31%)		
* ≥ 5*	89 (14%)	22 (17%)	67 (14%)		
* NA*	19 (3%)	4 (3%)	15 (3%)		
Katz ADL score (dependency score)					
* 0 = Total*	33 (6%)	12 (9%)	21 (4%)	Fisher’s exact	**0.016**
* 1–3 = High*	390 (63%)	74 (56%)	316 (66%)		
* 4–5 = Moderate*	161 (26%)	43 (33%)	118 (24%)		
* 6 = No dependency*	23 (4%)	2 (1%)	21 (4%)		
* NA*	9 (1%)	1 (1%)	8 (2%)		
Palliative care					
* No*	550 (89%)	107 (81%)	443 (92%)	Chi2	**< 0.001**
* Yes*	40 (7%)	19 (14%)	21 (4%)		
* NA*	26 (4%)	6 (5%)	20 (4%)		
Advance directives not to hospitalize					
* No*	546 (89%)	116 (88%)	430 (89%)	Fisher’s exact	0.078
* Yes*	7 (1%)	4 (3%)	3 (1%)		
* DNK*	63 (10%)	12 (9%)	51 (10%)		
**Characteristics of the nursing homes**					
French departments					
* Ariège (09)*	68 (11%)	22 (17%)	46 (10%)	Fisher’s exact	**0.039**
* Aveyron (12)*	26 (4%)	6 (5%)	20 (4%)		
* Haute-Garonne (31)*	204 (33%)	36 (27%)	168 (35%)		
* Gers (32)*	36 (6%)	5 (3%)	31 (6%)		
* Hérault (34)*	1 (1%)	1 (1%)	0 (0%)		
* Lot (46)*	23 (4%)	6 (5%)	17 (4%)		
* Hautes-Pyrénées (65)*	88 (14%)	26 (20%)	62 (13%)		
* Tarn (81)*	125 (20%)	21 (16%)	104 (21%)		
* Tarn-et-Garonne (82)*	45 (7%)	9 (6%)	36 (7%)		
PIU	118 (19%)	30 (23%)	88 (18%)	Chi2	0.239
Number of beds (mean, SD)	89 (35)	92 (36)	88 (34)	Mann-Whitney	0.112
Distance to the ED in kilometers (mean, SD)	17.52 (13.39)	16.56 (13.71)	17.78 (13.30)	Mann-Whitney	0.208
Coordinating physician	543 (88%)	119 (90%)	424 (88%)	Chi2	0.422
Geriatric nursing assistant	404 (66%)	87 (66%)	317 (65%)	Chi2	0.929
**Characteristics of the ED transfer**					
Period of transfer					
* January to March*	174 (28%)	33 (25%)	141 (29%)	Chi2	0.412
* April to June*	153 (25%)	35 (27%)	118 (25%)		
* July to September*	145 (24%)	37 (28%)	108 (22%)		
* October to December*	144 (23%)	27 (20%)	117 (24%)		
Post-transfer destination					
* Death*	6 (1%)	2 (1%)	4 (1%)	Chi2	**< 0.001**
* Back at nursing home*	284 (46%)	88 (67%)	196 (40%)		
* Hospitalization*	326 (53%)	42 (32%)	284 (59%)		

*SD* Standard deviation, *ED* Emergency department, *ADL* Activities of daily living, *NA* No answer, *DNK* Do not know, *PIU* Pharmacy for internal use

### Analysis of the main objective: comparison between the inappropriate and appropriate ED transfer groups

**Fig. 1:**
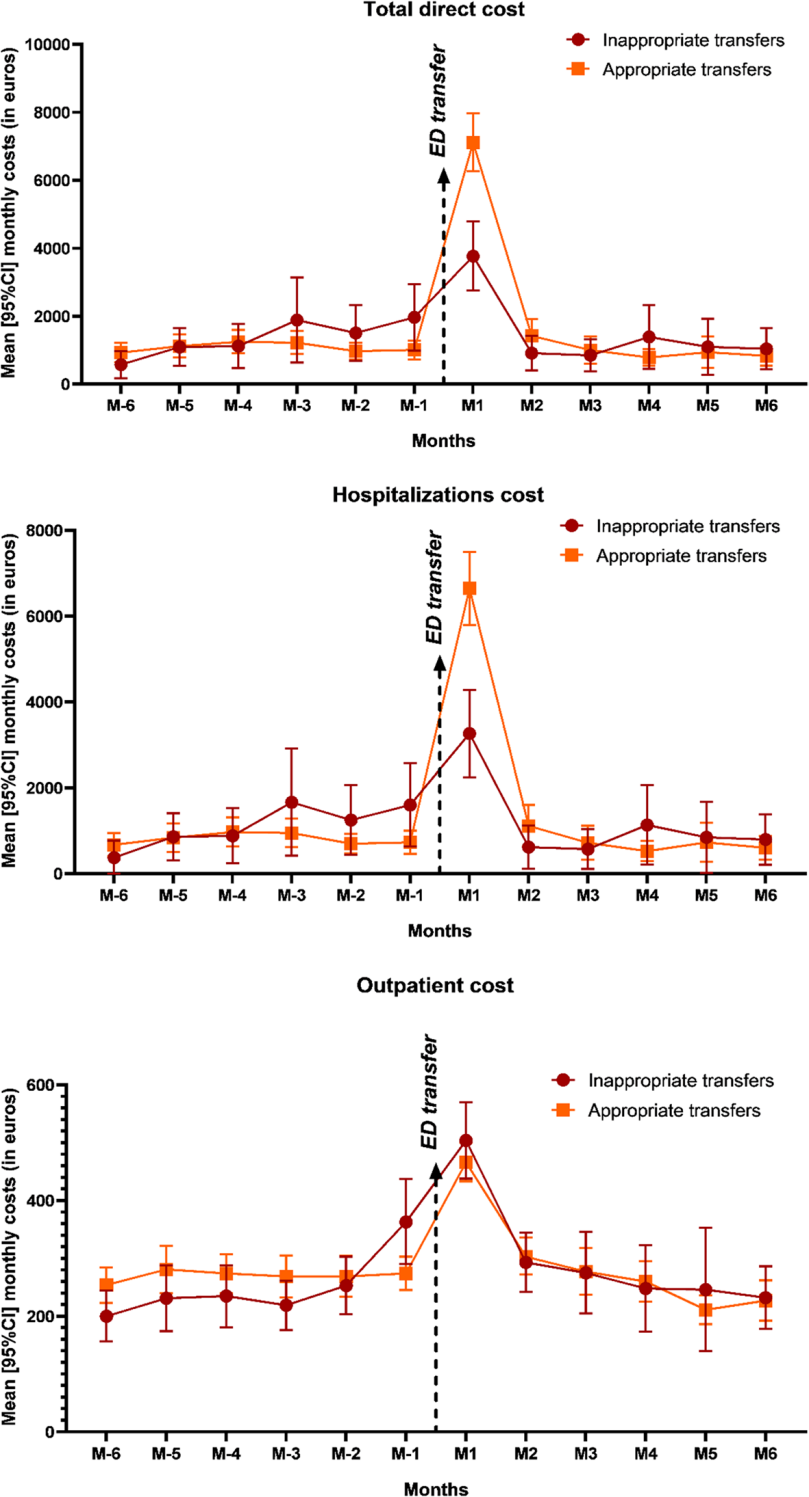
Mean [95%CI] monthly costs 6 months before and after transfer to the ED ED emergency department; 95%CI 95% Confidence intervals

Figure 1 presents total direct costs by month for both the appropriate and inappropriate ED transfer groups, then hospitalization and outpatient costs separately (see Supplementary Table S3 for details). In the 6 months before ED transfer, total direct costs on average amounted to 8,145€ for the inappropriate transfer group and 6,493€ for the appropriate transfer group. In the 6 months after ED transfer, they amounted on average to 9,050€ for the inappropriate transfer group and 12,094€ for the appropriate transfer group. Considering the whole period (6-month before and after ED transfer), these differences in average cost between inappropriate and appropriate transfer groups were non-significant (-1,392€, *p* = 0.57).

The largest part of costs after ED transfer occur during the first month (41.6% of the 6-month-post-transfer cost in the inappropriate transfer group and 58.8% in the appropriate transfer group). Regarding hospitalizations only, 6 months after ED transfer, costs amounted to 7,241€ and 10,346€ in the inappropriate and appropriate transfer groups, respectively. Most variation was due to conventional inpatient stays.

**Fig. 2:**
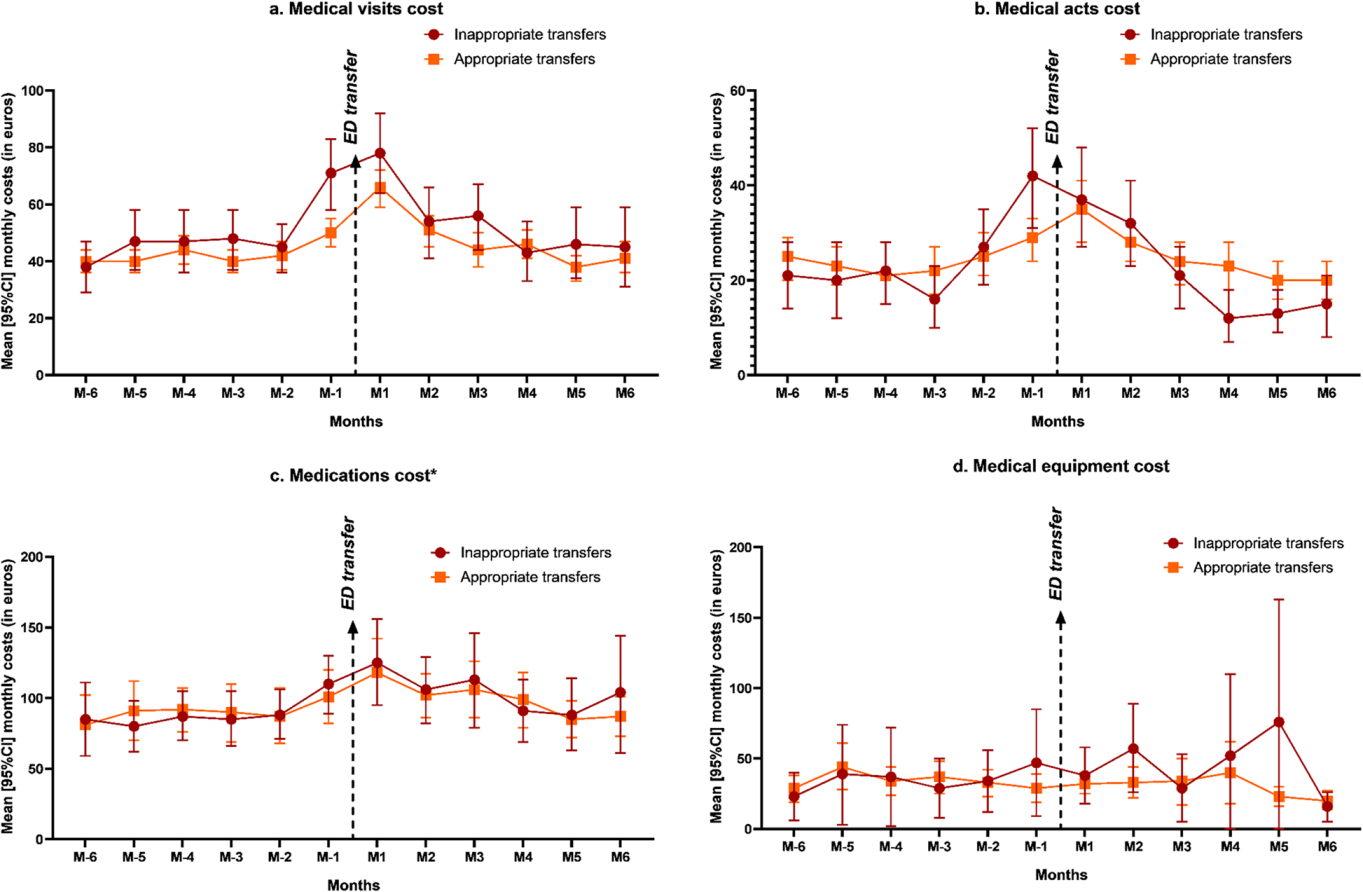
Mean [95%CI] monthly outpatient cost categories 6 months before and after transfer to the ED. **a**. Medical visits cost **b**. Medical acts cost **c**. Medications cost **d**. Medical equipment cost * Except for residents in nursing homes with a pharmacy for internal use. ED emergency department; 95%CI 95% Confidence intervals

For outpatient costs, the 6 months before ED transfer amounted to 1,501€ vs. 1,621€ and the 6 months after to 1,798€ vs. 1,744€ for the inappropriate and appropriate transfer groups, respectively. Costs were already increasing one month before ED transfer. Figure 2 presents different cost categories: medical visits, medical acts, medications and medical equipment. In the inappropriate transfer group, we can observe a larger costs’ increase starting the month before ED transfer compared to the appropriate transfer group. Considering the whole period, the differential cost between the two groups was significant only for medical visits (mean annual costs: 618€ in the inappropriate ED transfer group vs. 542€ in the appropriate ED transfer group, *p* = 0.007).

### Analysis of the secondary objective: multivariate analysis

**Fig. 3:**
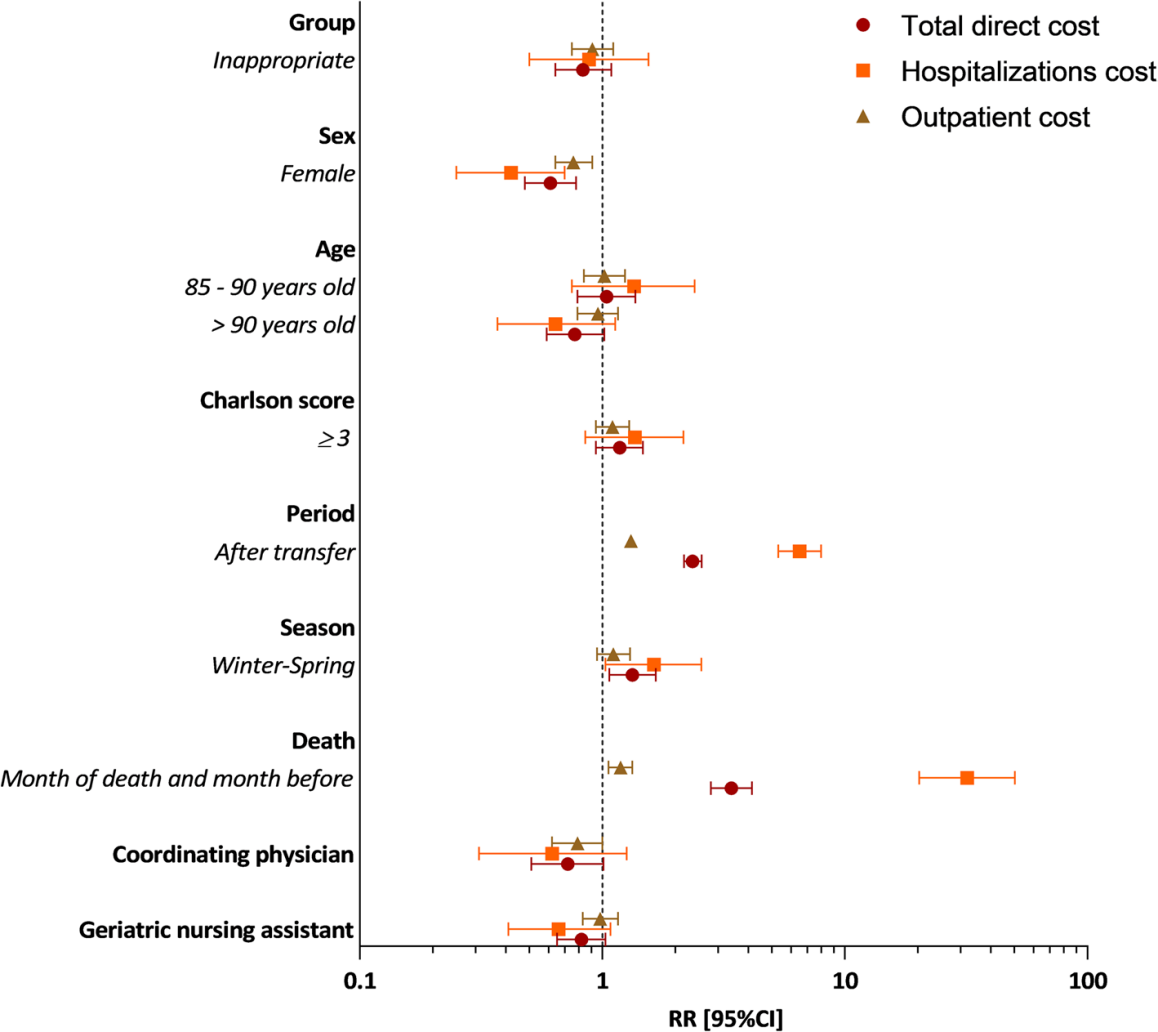
Predictors of total direct cost, hospitalizations cost and outpatient cost over a year (6 months before and after transfer to the ED). RR Relative risk; 95%CI 95% Confidence intervals; ED emergency department

Figure 3 shows the results of the adjusted GLMM for total direct costs and separately for hospitalization and outpatient costs (see details in Supplementary Table S4). During the follow-up year, adjusted for sex, age, Charlson score, period, season, death and NH human resources, total direct costs were 17% lower in the inappropriate group compared to the appropriate group (RR = 0.83; 95% CI 0.64–1.09, *p* = 0.19). On average, total direct costs were 39% lower for women compared to men (RR = 0.61; 95% CI 0.48–0.78, *p* < 0.001). Contrariwise, total direct costs were 33% higher when ED transfer occurred in seasons winter-spring (from January to June) than in seasons summer-autumn (RR = 1.33; 95% CI 1.07–1.66, *p* = 0.01), and were multiplied by 2.35 for the 6-month period after ED transfer (RR = 2.35; 95% CI 2.17–2.57, *p* < 0.001), and by 3.4 just the month before death (RR = 3.4; 95% CI 2.80–4.14, *p* < 0.001). Regarding hospitalizations cost, results were similar or even exacerbated for the 6-month period after ED transfer (RR = 6.51; 95% CI 5.32–7.97, *p* < 0.001) and death (RR = 31.89; 95% CI 20.27–50.19, *p* < 0.001). Regarding outpatient cares, on average, costs were 21% lower in NHs with coordinating physicians (RR = 0.79; 95% CI 0.62-1.00, *p* = 0.054).

**Fig. 4:**
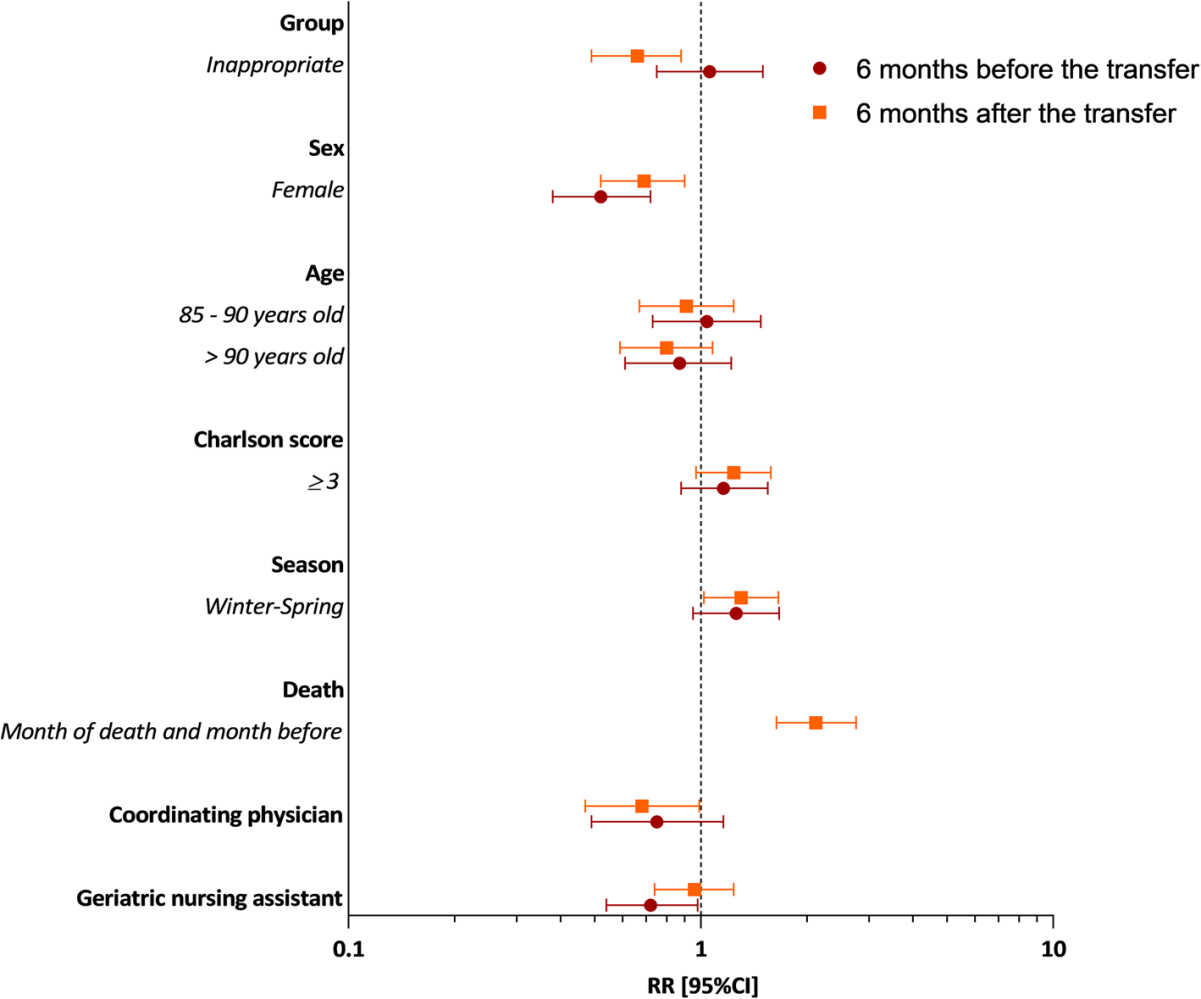
Predictors of total direct cost 6 months before and 6 months after transfer to the ED. RR Relative risk; 95%CI 95% Confidence intervals; ED emergency department

Figure 4 shows the results of the adjusted GLMM, for total direct cost over two separate periods: 6 months before and 6 months after ED transfer (see details in Supplementary Table S5). Six months before, total direct costs were 6% higher in the inappropriate group compared to the appropriate group, but non-significant (RR = 1.06; 95% CI 0.75–1.50, *p* = 0.736). On average, costs were 48% lower for women compared to men (RR = 0.52; 95% CI 0.38–0.72, *p* < 0.001), 25% lower in NHs with coordinating physicians compared to NHs without (RR = 0.75; 95% CI 0.49–1.16, *p* = 0.2021) and 28% lower in NHs with geriatric nursing assistants compared to NHs without (RR = 0.72; 95% CI 0.54–0.98, *p* = 0.0345). Six months after, total direct costs were lower in the inappropriate group (RR = 0.66; 95% CI 0.49–0.88, *p* = 0.006), for women (RR = 0.69; 95% CI 0.52–0.90, *p* = 0.006) and in NHs with coordinating physicians (RR = 0.68. 95% CI 0.47–0.99, *p* = 0.047).

## Discussion

The principal findings of this study show that total costs are higher after the transfer to the ED compared to before, whatever the transfer is appropriate or inappropriate, and mainly in the first month following the transfer. Moreover, when the transfer to the ED is inappropriate, there were an increase in cost of care 6-months before ED transfer, and we observed higher outpatient costs during this period compared with the appropriate transfer group (despite a small (6%) and non-significant difference between our two groups). Our first hypothesis that inappropriate transfer to the ED could be associated with increased expenditures 6-months before and after the transfer (in- and outpatient costs) is not confirmed by our results (non-significant).

To our knowledge, there is no study aiming to estimate the extra-cost 6-months before and after the ED transfer, taking into account the overall costs (out- and inpatient) due to inappropriate ED transfer. Several studies aiming to estimate cost of inappropriate ED transfer and potentially avoidable hospitalization, but considering only inpatient costs. These studies showed an inpatient costs increase in case of inappropriate ED admissions or avoidable hospitalizations [17, 18], but these inpatient costs are still lower than those for appropriate hospitalizations, when there is a comparison [13]. For example, in 2015, an American study explained that a significant proportion of Medicare NH patients were transferred to the ED for ambulatory care-sensitive conditions (ACSC) [28]. This was associated with higher healthcare utilization and ED costs (mean ED costs/episode of care: $401 vs. $294 for ACSC patients compared to non-ACSC patients), but lower hospitalization costs (mean hospitalization costs/episode of care: $8,356 vs. $10,226, *p* < 0,001).

We found a similar hospitalization cost difference in our study (but non-significant): the increase in total costs is higher in the appropriate transfer group than in the inappropriate one. However, hospitalization costs represent the largest part of total costs (between 80% and 86% after ED transfer). So, this result can be explained because patients in the appropriate transfer group are hospitalized more often and for a longer period after the ED visit than patients in the inappropriate transfer group.

During the 6-month period before ED transfer, the higher outpatient costs were unexpected in the inappropriate transfer group and require further exploration, even if non-significant results were observed. It may indicate a suboptimal care pathway. Some nonspecific symptoms, like confusion, disorientation, agitation or complaints of pain, could lead to misdiagnosis with recurrent medical exams, medical visits, ED transfers and hospitalizations. It is common in older people, especially in case of mild cognitive impairment or dementia; it has been reported that dementia increases healthcare utilization and expenditures [29–32]. Therefore, it is important to coordinate care and to take into account as a priority the needs and preferences of older people as proposed by the WHO guidelines on Integrated Care for Older People (ICOPE) [33].

Concerning factors associated with healthcare utilization’s costs among NH residents, total costs were significantly lower for women. We know that women have better health behavior, with better medical follow-up. They live longer in good health and they are less expensive for the healthcare system [34, 35]. Moreover, in our study, men had more comorbidities (57% with Charlson score ≥ 3 vs. 38.6% for women), resulting in additional healthcare expenditures. Second, death was associated with a significant increase in costs, mainly due to hospital costs. The healthcare utilization is often high in the last months of life with many hospitalizations [36]. However, substantial savings are possible with a specific end-of-life program in NHs (comfort measures and limited medical intervention), thus decreasing hospitalizations and associated inpatient costs [37]. Third, costs were higher during the first half of the year (winter and spring). We can imagine that winter epidemics (influenza, bronchitis, gastroenteritis) cause an increase in healthcare utilization (medical visits, medications) and related costs. Indeed, epidemic peaks are more frequently observed at the beginning of the year. This was indeed the case for our study period, with an epidemic peak of influenza in January-February 2016 and 2017 [38].

This study also highlighted NH organization’s impact on healthcare costs. Before ED transfer, the presence of a geriatric nursing assistant in NHs significantly reduces costs and after ED transfer, this is the presence of a coordinating physician. In France, geriatric nursing assistants play a preventive and alert role with dementia patients: they identify potential decompensation and anticipate their care. Thus, we can speculate that this helps avoid the aggravation of symptoms and the costs of care it entails. Coordinating physicians are family doctors whose training in geriatrics includes 70 h of theoretical training and 70 h of practical training. They are present around 1 to 2 days per week in the NH and they are in charge of the comprehensive geriatric assessment of the resident and the coordination of the care and staff in the NH. Due to this, the coordinating physician improves interactions between the different health professionals, for optimized and less expensive patient care. NH organizational characteristics may thus affect individual healthcare consumption and costs. For example, several studies from the USA show that the use of advanced practice nurses reduces preventable hospitalizations [39, 40]. In summary, better access to on-site evaluation could have favorable effects on healthcare utilization and expenditures [28, 41]. This result is important for policymakers when considering resources provided to NH staff to take care of, treat and ultimately avoid the ED transfer of NH residents.

This study has several strengths. This is the first cost analysis to study healthcare expenditures before and after the inappropriate ED transfer, taking into account the overall costs (out- and inpatient costs). Moreover, this analysis used data from the FHI database, which is an accurate data source to gather healthcare consumption. It was a real-life study because all patients from NHs were included, with no other selection criteria. Therefore, our observed results reflected reality [42–44].

However, this study also has some limitations. First, the health economic analysis was limited to patients covered by the general worker’s regimen and farmer’s regimen with the use of the FHI database. The presence of 60% of the FINE population in the FHI database, instead of the 80% expected, could be explained by a different distribution of regimens in NHs or a healthcare utilization in another region. Furthermore, inpatient stays were not valued using the French disease-related groups of the French hospital-discharge database (PMSI), but the billing data used was close enough to real costs. Another limitation is that healthcare costs may vary between NHs for several reasons, and the total cost may therefore be underestimated. The main reason is the presence or absence of a PIU. In this study, 19% of NHs have a PIU, which means that drugs dispensed by this PIU are not specifically reimbursed individually for the patient but for the whole NH [45], and only individual reimbursements for the patient are available in the FHI database. Moreover, costs may be underestimated because of the absence of accommodation and food costs, as well as the cost of burden staff, formal and informal cares. These data are not available in the FHI database. Formal care costs in NHs increase according to the dependence level of the patient, and even if the informal care cost is not as high as in community-dwelling patients, it can be a significant part of the total healthcare cost of NH residents [46].

## Conclusions

To conclude, we have not shown any significant increase in healthcare expenditure with inappropriate ED transfer. However, actions could be considered to prevent these inappropriate transfers from NH, with a possible financial impact on the healthcare system; how to better allocate these resources, e.g. to fund interventions aimed at improving primary care access, such as implementing ICOPE in long-term care facilities. In addition, support for NH staff and better pathways of care seem necessary to reduce healthcare expenditures in NH residents.

### Supplementary Information

**Supplementary Material 1.**

## Data Availability

The datasets used and analysed during the current study are available from the corresponding author on reasonable request.

## Abbreviations

ACSC: Ambulatory care-sensitive conditions
ED: Emergency department
FHI: French Health Insurance
GLMM: Generalized linear mixed model
NH: Nursing home
PIU: Pharmacy for internal use
