# Supplementary material for: Cost of care pathways before and after appropriate and inappropriate transfers to the emergency department among nursing home residents: results from the FINE study
Source: BMC Geriatr. 2024 Apr 19;24:353. doi: 10.1186/s12877-024-04946-x (PMC11027376; doi:10.1186/s12877-024-04946-x)
Supplement: Supplementary file 1 — Supplementary Material 1. [file 12877_2024_4946_MOESM1_ESM.docx]

**Supplementary S1. Flow chart**


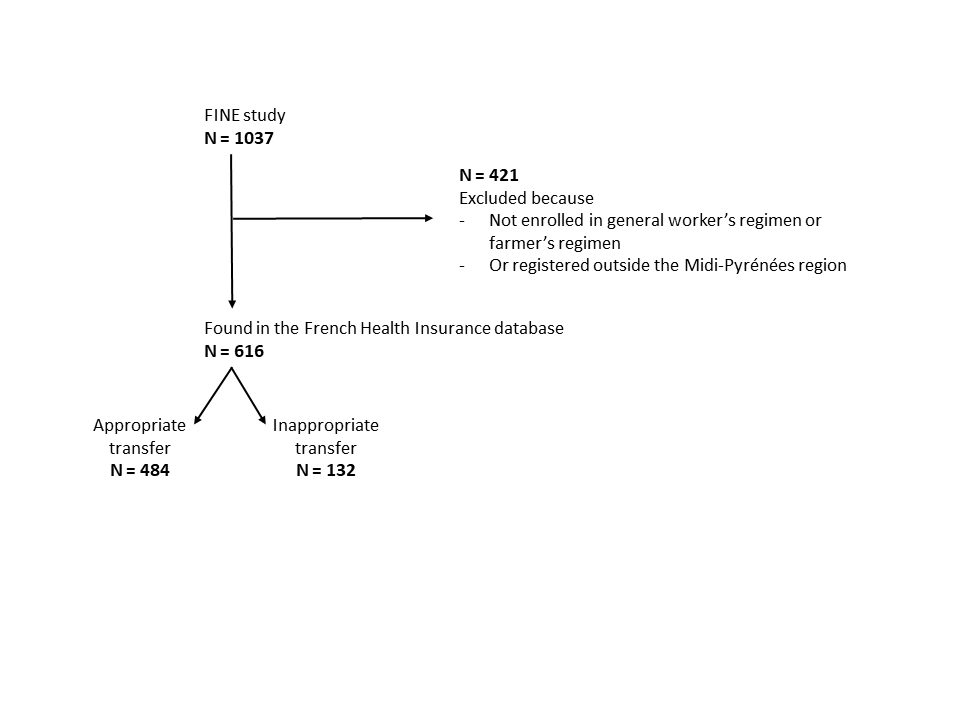


**Supplementary S2. Characteristics comparison between the French Health Insurance (FHI) population and the rest of FINE population (except FHI)**

|  |  | FHI **N =616** | FINE except FHI **N = 421** | Statistical test | p-value |
| --- | --- | --- | --- | --- | --- |
| **Group** | Inappropriate | 132 (21.43%) | 88 (20.90%) | Chi2 | 0.84 |
|  | Appropriate | 484 (78.57%) | 333 (79.10%) |  |  |
|  |  |  |  |  |  |
| **INAPPROPRIATE TRANSFER** | | **N = 132** | **N = 88** |  |  |
| **Characteristics of the residents** | |  |  |  |  |
| Female |  | 86 (65%) | 53 (60%) | Chi2 | 0.46 |
| Age (mean, SD) |  | 85.81 (7.97) | 87.61 (7.37) | Mann-Whitney | **0.03** |
| Charlson score (mean, SD) | | 2.68 (2.24) | 2.79 (2.20) | Mann-Whitney | 0.78 |
| Katz ADL score (mean, SD) | | 2.27 (1.79) | 1.88 (1.78) | Mann-Whitney | 0.30 |
| Palliative care |  | 19 (14%) | 13 (14%) | Chi2 | 0.94 |
| Advance directives not to hospitalize | | 4 (3%) | 3 (3%) | Chi2 | 0.88 |
| **Characteristics of the nursing homes** | |  |  |  |  |
| PIU |  | 30 (23%) | 13 (15%) | Chi2 | 0.14 |
| Number of beds (mean, SD) | | 92 (36) | 87 (40) | Mann-Whitney | 0.43 |
| Distance to the ED in kilometers (mean, SD) | | 16.56 (13.71) | 17.27 (14.78) | Mann-Whitney | 0.75 |
| Coordinating physician | | 119 (90%) | 79 (90%) | Chi2 | 0.93 |
| Geriatric nursing assistant | | 87 (66%) | 53 (60%) | Chi2 | 0.39 |
| **Characteristics of the ED transfer** | |  |  |  |  |
| Period of transfer | *January to March* | 33 (25%) | 22 (25%) | Chi2 | 0.25 |
|  | *April to June* | 35 (27%) | 18 (20%) |  |  |
|  | *July to September* | 37 (28%) | 20 (23%) |  |  |
|  | *October to December* | 27 (20%) | 28 (32%) |  |  |
| Post-transfer destination | *Death* | 2 (1%) | 0 (0%) | Chi2 | 0.37 |
|  | *Back at nursing home* | 88 (67%) | 55 (62%) |  |  |
|  | *Hospitalization* | 42 (32%) | 33 (38%) |  |  |
|  |  |  |  |  |  |
| **APPROPRIATE TRANSFER** | | **N = 484** | **N = 333** |  |  |
| **Characteristics of the residents** | |  |  |  |  |
| Female |  | 339 (70%) | 231 (69%) | Chi2 | 0.84 |
| Age (mean, SD) |  | 87.34 (7.06) | 88.46 (6.41) | Mann-Whitney | 0.06 |
| Charlson score (mean, SD) | | 2.67 (1.91) | 2.59 (2.04) | Mann-Whitney | 0.57 |
| Katz ADL score (mean, SD) | | 2.48 (1.62) | 2.58 (1.62) | Mann-Whitney | 0.08 |
| Palliative care |  | 21 (4%) | 17 (5%) | Chi2 | 0.61 |
| Advance directives not to hospitalize | | 3 (1%) | 5 (2%) | Chi2 | 0.21 |
| **Characteristics of the nursing homes** | |  |  |  |  |
| PIU |  | 88 (18%) | 65 (20%) | Chi2 | 0.63 |
| Number of bed (mean, SD) | | 88 (34) | 89 (34) | Mann-Whitney | 0.43 |
| Distance to the ED in kilometers (mean, SD) | | 17.78 (13.30) | 18.93 (15.27) | Mann-Whitney | 0.75 |
| Coordinating physician | | 424 (88%) | 285 (86%) | Chi2 | 0.40 |
| Geriatric nursing assistant | | 317 (65%) | 232 (70%) | Chi2 | 0.21 |
| **Characteristics of the ED transfer** | |  |  |  |  |
| Period of transfer | *January to March* | 141 (29%) | 89 (27%) | Chi2 | 0.87 |
|  | *April to June* | 118 (25%) | 82 (25%) |  |  |
|  | *July to September* | 108 (22%) | 75 (22%) |  |  |
|  | *October to December* | 117 (24%) | 87 (26%) |  |  |
| Post-transfer destination | *Death* | 4 (1%) | 2 (1%) | Chi2 | 0.63 |
|  | *Back at nursing home* | 196 (40%) | 125 (37%) |  |  |
|  | *Hospitalization* | 284 (59%) | 206 (62%) |  |  |

SD standard deviation; ED emergency department; ADL activities of daily living; PIU pharmacy for internal use

**Supplementary S3. Mean [95%CI] monthly costs 6 months before and after transfer to the ED**

|  | **Total direct cost** | | | | | **Hospitalizations cost** | | | | |
| --- | --- | --- | --- | --- | --- | --- | --- | --- | --- | --- |
| Months | Inappropriate transfer | | Appropriate  transfer | | p-value  (Wilcoxon test) | Inappropriate transfer | | Appropriate  transfer | | p-value  (Wilcoxon test) |
|  |  |  |  |  |  |  |  |  |  |  |
| M-6 | 576 | [178;973] | 928 | [649;1208] | 0.57 | 375 | [-22;772] | 675 | [400;950] | 0.6 |
| M-5 | 1091 | [530;1653] | 1122 | [779;1466] |  | 860 | [313;1407] | 842 | [509;1174] |  |
| M-4 | 1120 | [466;1774] | 1247 | [900;1595] |  | 885 | [241;1530] | 973 | [636;1311] |  |
| M-3 | 1886 | [634;3139] | 1221 | [884;1558] |  | 1668 | [418;2917] | 952 | [625;1279] |  |
| M-2 | 1507 | [682;2332] | 969 | [723;1216] |  | 1253 | [437;2069] | 700 | [466;935] |  |
| M-1 | 1965 | [984;2946] | 1006 | [726;1286] |  | 1602 | [632;2571] | 732 | [462;1003] |  |
| M1 | 3768 | [2748;4787] | 7112 | [6261;7963] |  | 3263 | [2247;4278] | 6645 | [5797;7493] |  |
| M2 | 912 | [406;1418] | 1422 | [931;1913] |  | 619 | [114;1125] | 1117 | [634;1601] |  |
| M3 | 848 | [375;1321] | 1001 | [598;1404] |  | 573 | [109;1037] | 723 | [330;1117] |  |
| M4 | 1387 | [448;2327] | 787 | [550;1025] |  | 1139 | [214;2064] | 527 | [294;760] |  |
| M5 | 1096 | [270;1922] | 940 | [484;1396] |  | 850 | [25;1675] | 729 | [275;1183] |  |
| M6 | 1039 | [429;1648] | 832 | [547;1117] |  | 797 | [205;1388] | 605 | [325;885] |  |
|  |  |  |  |  |  |  |  |  |  |  |
|  | **Outpatient cost** | | | | | **Medical visits cost** | | | | |
| Months | Inappropriate transfer | | Appropriate  transfer | | p-value  (Wilcoxon test) | Inappropriate transfer | | Appropriate  transfer | | p-value  (Wilcoxon test) |
|  |  |  |  |  |  |  |  |  |  |  |
| M-6 | 200 | [156;244] | 254 | [223;284] | 0.37 | 38 | [29;47] | 40 | [36;44] | 0.007 |
| M-5 | 231 | [174;288] | 281 | [240;322] |  | 47 | [37;58] | 40 | [36;44] |  |
| M-4 | 235 | [181;288] | 274 | [240;307] |  | 47 | [36;58] | 44 | [39;49] |  |
| M-3 | 219 | [176;261] | 269 | [232;305] |  | 48 | [37;58] | 40 | [36;44] |  |
| M-2 | 253 | [203;303] | 269 | [234;304] |  | 45 | [36;53] | 42 | [37;47] |  |
| M-1 | 363 | [290;437] | 274 | [245;303] |  | 71 | [58;83] | 50 | [45;55] |  |
| M1 | 504 | [438;570] | 466 | [433;500] |  | 78 | [64;92] | 66 | [59;72] |  |
| M2 | 293 | [242;344] | 303 | [272;336] |  | 54 | [41;66] | 51 | [45;56] |  |
| M3 | 275 | [205;346] | 277 | [237;318] |  | 56 | [44;67] | 44 | [38;50] |  |
| M4 | 248 | [173;323] | 260 | [225;295] |  | 43 | [33;54] | 46 | [41;51] |  |
| M5 | 246 | [140;353] | 211 | [186;236] |  | 46 | [34;59] | 38 | [33;42] |  |
| M6 | 232 | [178;286] | 227 | [192;262] |  | 45 | [31;59] | 41 | [36;47] |  |
|  |  |  |  |  |  |  |  |  |  |  |
|  | **Medical acts cost** | | | | | **Medications cost** **^a^** | | | | |
| Months | Inappropriate transfer | | Appropriate  transfer | | p-value  (Wilcoxon test) | Inappropriate transfer | | Appropriate  transfer | | p-value  (Wilcoxon test) |
|  |  |  |  |  |  |  |  |  |  |  |
| M-6 | 21 | [14;28] | 25 | [20;29] | 0.3 | 85 | [59;111] | 81 | [59;102] | 0.56 |
| M-5 | 20 | [12;28] | 23 | [19;27] |  | 80 | [62;98] | 91 | [70;112] |  |
| M-4 | 22 | [15;28] | 21 | [15;28] |  | 87 | [70;105] | 92 | [76;107] |  |
| M-3 | 16 | [10;23] | 22 | [17;27] |  | 85 | [66;105] | 90 | [69;110] |  |
| M-2 | 27 | [19;35] | 25 | [21;30] |  | 88 | [71;106] | 87 | [68;107] |  |
| M-1 | 42 | [31;52] | 29 | [24;33] |  | 110 | [89;130] | 101 | [82;120] |  |
| M1 | 37 | [27;48] | 35 | [28;41] |  | 125 | [95;156] | 118 | [95;142] |  |
| M2 | 32 | [23;41] | 28 | [24;32] |  | 106 | [82;129] | 102 | [86;117] |  |
| M3 | 21 | [14;27] | 24 | [19;28] |  | 113 | [79;146] | 106 | [86;126] |  |
| M4 | 12 | [7;18] | 23 | [18;28] |  | 91 | [69;113] | 99 | [79;118] |  |
| M5 | 13 | [9;18] | 20 | [16;24] |  | 88 | [63;114] | 85 | [72;98] |  |
| M6 | 15 | [8;21] | 20 | [16;24] |  | 104 | [61;144] | 87 | [73;101] |  |
|  |  |  |  |  |  |  |  |  |  |  |
|  |  |  |  |  |  |  |  |  |  |  |
|  |  |  |  |  |  |  |  |  |  |  |
|  |  |  |  |  |  |  |  |  |  |  |
|  |  |  |  |  |  |  |  |  |  |  |
|  |  |  |  |  |  |  |  |  |  |  |
|  | **Medical equipment cost** | | | | | **Transportation cost** | | | | |
| Months | Inappropriate transfer | | Appropriate  transfer | | p-value  (Wilcoxon test) | Inappropriate transfer | | Appropriate  transfer | | p-value  (Wilcoxon test) |
|  |  |  |  |  |  |  |  |  |  |  |
| M-6 | 23 | [6;40] | 29 | [19;38] | 0.3 | 31 | [17;44] | 53 | [39;67] | 0.2 |
| M-5 | 39 | [3;74] | 44 | [28;61] |  | 39 | [20;58] | 57 | [36;78] |  |
| M-4 | 37 | [2;72] | 34 | [24;44] |  | 38 | [20;56] | 64 | [42;87] |  |
| M-3 | 29 | [8;50] | 37 | [25;48] |  | 36 | [24;49] | 69 | [46;92] |  |
| M-2 | 34 | [12;56] | 33 | [23;42] |  | 56 | [37;75] | 75 | [51;100] |  |
| M-1 | 47 | [9;85] | 29 | [19;39] |  | 98 | [63;134] | 65 | [50;80] |  |
| M1 | 38 | [18;58] | 32 | [25;40] |  | 242 | [206;277] | 220 | [204;237] |  |
| M2 | 57 | [26;89] | 33 | [22;44] |  | 55 | [38;72] | 88 | [71;104] |  |
| M3 | 29 | [5;53] | 34 | [17;50] |  | 69 | [42;97] | 71 | [45;97] |  |
| M4 | 52 | [-7;110] | 40 | [18;62] |  | 59 | [33;85] | 55 | [40;70] |  |
| M5 | 76 | [-11;163] | 23 | [16;30] |  | 32 | [16;48] | 44 | [28;61] |  |
| M6 | 16 | [5;26] | 20 | [14;27] |  | 67 | [34;101] | 59 | [31;86] |  |

^a^ Except for residents in nursing homes with a pharmacy for internal use

**Supplementary S4. Predictors of total direct cost, hospitalizations cost and outpatient cost over a year (6 months before and after transfer to the ED): Results from generalized linear mixed model (GLMM)**

|  |  | | **Total direct costs** | | | **Hospitalizations cost** | | | **Outpatient cost** | | | |
| --- | --- | --- | --- | --- | --- | --- | --- | --- | --- | --- | --- | --- |
|  |  | | RR | 95% CI | p-value | RR | 95% CI | p-value | RR | 95% CI | p-value |  |
| **Group** | *Appropriate* | | 1 |  |  | 1 |  |  | 1 |  |  |  |
|  | *Inappropriate* | | 0.83 | [0.64-1.09] | 0.19 | 0.88 | [0.50-1.55] | 0.66 | 0.91 | [0.75-1.11] | 0.363 |  |
|  |  | |  |  |  |  |  |  |  |  |  |  |
| **Sex** | *Male* | | 1 |  |  | 1 |  |  | 1 |  |  |  |
|  | *Female* | | 0.61 | [0.48-0.78] | **<0.001** | 0.42 | [0.25-0.70] | **<0.001** | 0.76 | [0.64-0.91] | **0.002** |  |
|  |  | |  |  |  |  |  |  |  |  |  |  |
| **Age** | *< 85 years old* | | 1 |  |  | 1 |  |  | 1 |  |  |  |
|  | *85 - 90 years old* | | 1.04 | [0.79-1.37] | 0.795 | 1.35 | [0.75-2.40] | 0.267 | 1.02 | [0.84-1.24] | 0.855 |  |
|  | *> 90 years old* | | 0.77 | [0.59-1.02] | 0.063 | 0.64 | [0.37-1.13] | 0.128 | 0.96 | [0.79-1.16] | 0.678 |  |
|  |  | |  |  |  |  |  |  |  |  |  |  |
| **Charlson score** | *0-2* | | 1 |  |  | 1 |  |  | 1 |  |  |  |
|  | *3 +* | | 1.18 | [0.94-1.47] | 0.148 | 1.36 | [0.85-2.16] | 0.206 | 1.1 | [0.94-1.29] | 0.252 |  |
|  |  | |  |  |  |  |  |  |  |  |  |  |
| **Period** | *Before transfer* | | 1 |  |  | 1 |  |  | 1 |  |  |  |
|  | *After transfer* | | 2.35 | [2.17-2.57] | **<0.001** | 6.51 | [5.32-7.97] | **<0.001** | 1.31 | [1.24-1.38] | **<0.001** |  |
|  |  | |  |  |  |  |  |  |  |  |  |  |
| **Season** | *Summer-Autumn* | | 1 |  |  | 1 |  |  | 1 |  |  |  |
|  | *Winter-Spring* | | 1.33 | [1.07-1.66] | **0.01** | 1.63 | [1.03-2.56] | **0.036** | 1.11 | [0.95-1.30] | 0.193 |  |
|  |  | |  |  |  |  |  |  |  |  |  |  |
| **Death ^a^** | No | | 1 |  |  | 1 |  |  | 1 |  |  |  |
|  | Yes | | 3.4 | [2.80-4.14] | **<0.001** | 31.89 | [20.27-50.19] | **<0.001** | 1.19 | [1.06-1.33] | **0.003** |  |
|  |  | |  |  |  |  |  |  |  |  |  |  |
| **Coordinating physician** | | No | 1 |  |  | 1 |  |  | 1 |  |  |  |
|  |  | Yes | 0.72 | [0.51-1.01] | 0.059 | 0.62 | [0.31-1.26] | 0.199 | 0.79 | [0.62-1.00] | 0.054 |  |
| **Geriatric nursing assistant** | | No | 1 |  |  | 1 |  |  | 1 |  |  |  |
|  |  | Yes | 0.82 | [0.65-1.03] | 0.089 | 0.66 | [0.41-1.08] | 0.095 | 0.98 | [0.83-1.16] | 0.828 |  |

^a^ Month of death and month before; RR relative risk; CI confidence interval

**Supplementary S5. Predictors of total direct cost 6 months before and 6 months after transfer to the ED: Results from generalized linear mixed model (GLMM)**

|  |  | **Before transfer** | | | **After transfer** | | | |
| --- | --- | --- | --- | --- | --- | --- | --- | --- |
|  |  | RR | 95%CI | p-value | RR | 95%CI | p-value |  |
|  | *Appropriate* | 1 |  |  | 1 |  |  |  |
| **Group** | *Inappropriate* | 1.06 | [0.75-1.50] | 0.736 | 0.66 | [0.49-0.88] | **0.006** |  |
|  |  |  |  |  |  |  |  |  |
|  | *Male* | 1 |  |  | 1 |  |  |  |
| **Sex** | *Female* | 0.52 | [0.38-0.72] | **<0.001** | 0.69 | [0.52-0.90] | **0.006** |  |
|  |  |  |  |  |  |  |  |  |
| **Age** | *< 85 years old* | 1 |  |  | 1 |  |  |  |
|  | *85 - 90 years old* | 1.04 | [0.73-1.48] | 0.8379 | 0.91 | [0.67-1.24] | 0.549 |  |
|  | *> 90 years old* | 0.87 | [0.61-1.22] | 0.4149 | 0.8 | [0.59-1.08] | 0.138 |  |
|  |  |  |  |  |  |  |  |  |
| **Charlson score** | *0-2* | 1 |  |  | 1 |  |  |  |
|  | *3 +* | 1.16 | [0.88-1.55] | 0.2961 | 1.24 | [0.97-1.58] | 0.093 |  |
|  |  |  |  |  |  |  |  |  |
| **Season** | *Summer-Autumn* | 1 |  |  | 1 |  |  |  |
|  | *Winter-Spring* | 1.26 | [0.95-1.67] | 0.1092 | 1.3 | [1.02-1.66] | **0.033** |  |
|  |  |  |  |  |  |  |  |  |
| **Death** **^a^** | No |  |  |  | 1 |  |  |  |
|  | Yes |  |  |  | 2.12 | [1.64-2.76] | **<0.001** |  |
|  |  |  |  |  |  |  |  |  |
| **Coordinating physician** | No | 1 |  |  | 1 |  |  |  |
|  | Yes | 0.75 | [0.49-1.16] | 0.2021 | 0.68 | [0.47-0.99] | **0.047** |  |
| **Geriatric nursing assistant** | No | 1 |  |  | 1 |  |  |  |
|  | Yes | 0.72 | [0.54-0.98] | **0.0345** | 0.96 | [0.74-1.24] | 0.729 |  |

^a^ Month of death and month before; RR relative risk; CI confidence interval
